# Plant Species-Dependent Increased Abundance and Diversity of IncP-1 Plasmids in the Rhizosphere: New Insights Into Their Role and Ecology

**DOI:** 10.3389/fmicb.2020.590776

**Published:** 2020-11-27

**Authors:** Masaki Shintani, Eman Nour, Tarek Elsayed, Khald Blau, Inessa Wall, Sven Jechalke, Cathrin Spröer, Boyke Bunk, Jörg Overmann, Kornelia Smalla

**Affiliations:** ^1^Department of Engineering, Graduate School of Integrated Science and Technology, Shizuoka University, Hamamatsu, Japan; ^2^Department of Environment and Energy Systems, Graduate School of Science and Technology, Shizuoka University, Hamamatsu, Japan; ^3^Green Energy Research Division, Research Institute of Green Science and Technology, Shizuoka University, Hamamatsu, Japan; ^4^Julius Kühn Institute (JKI) – Federal Research Centre for Cultivated Plants, Institute for Epidemiology and Pathogen Diagnostics, Braunschweig, Germany; ^5^Department Microbial Ecology and Diversity Research, Leibniz Institute DSMZ-German Collection of Microorganisms and Cell Cultures, Braunschweig, Germany

**Keywords:** lettuce-, tomato-, and potato rhizosphere, *korB* qPCR, IncP-1 plasmid, PacBio sequencing

## Abstract

IncP-1 plasmids, first isolated from clinical specimens (R751, RP4), are recognized as important vectors spreading antibiotic resistance genes. The abundance of IncP-1 plasmids in the environment, previously reported, suggested a correlation with anthropogenic pollution. Unexpectedly, qPCR-based detection of IncP-1 plasmids revealed also an increased relative abundance of IncP-1 plasmids in total community DNA from the rhizosphere of lettuce and tomato plants grown in non-polluted soil along with plant age. Here we report the successful isolation of IncP-1 plasmids by exploiting their ability to mobilize plasmid pSM1890. IncP-1 plasmids were captured from the rhizosphere but not from bulk soil, and a high diversity was revealed by sequencing 14 different plasmids that were assigned to IncP-1β, δ, and ε subgroups. Although backbone genes were highly conserved and mobile elements or remnants as Tn*501*, IS*1071*, Tn*402*, or class 1 integron were carried by 13 of the sequenced IncP-1 plasmids, no antibiotic resistance genes were found. Instead, seven plasmids had a *mer* operon with Tn*501*-like transposon and five plasmids contained putative metabolic gene clusters linked to these mobile elements. In-depth sequence comparisons with previously known plasmids indicate that the IncP-1 plasmids captured from the rhizosphere are archetypes of those found in clinical isolates. Our findings that IncP-1 plasmids do not always carry accessory genes in unpolluted rhizospheres are important to understand the ecology and role of the IncP-1 plasmids in the natural environment.

## Introduction

Plasmids are self-replicating extrachromosomal DNA molecules and some of them are mobile genetic elements (MGEs) mediating the exchange of genetic information between bacterial cells via conjugation, mobilization, or transformation. Broad host range plasmids belonging to the ncompatibility (Inc) groups P/P-1, W, N, Q/P-4, and U/P-6 as well as the more recently proposed PromA (Van der Auwera et al., 2009) are of great interest, owing to their ability to transfer between, and maintain themselves in, bacteria of different taxa (Dröge et al., 2000). Among these, plasmids affiliated to IncP-1 group were shown to efficiently transfer under various environmental conditions (Heuer and Smalla, 2012; Shintani et al., 2014; Klümper et al., 2015). The first IncP-1 plasmids R751 and RP4 were isolated from clinical specimens (Datta et al., 1971; Jobanputra and Datta, 1974). Later, IncP-1 plasmids were reported from diverse environments, e.g., sewage, marine sediment, manure, biofilters, and rhizosphere that were affected by anthropogenic pollutants (Dahlberg et al., 1997; Schlüter et al., 2007; Binh et al., 2008; Bahl et al., 2009; Gomes et al., 2010; Heuer et al., 2012; Brown et al., 2013; Jechalke et al., 2013; Dealtry et al., 2014; Wolters et al., 2015). A wide variety of adaptive traits conferring resistance to antibiotics, disinfectants, heavy metals, and/or degradation of xenobiotics was found as accessory genes in the insertion hot spots of IncP-1 plasmids (Hsu and Bartha, 1979; Top and Springael, 2003; Smets and Barkay, 2005). IncP-1 plasmids were so far mainly reported from polluted environments (Schlüter et al., 2003; Tauch et al., 2003; Heuer et al., 2004; Tennstedt et al., 2005; Kamachi et al., 2006; Smalla et al., 2006). It was assumed that bacterial populations carrying IncP-1 plasmids encoding adaptive traits either increased in abundance due to the plasmid-conferred selective advantages or newly evolved in the environments under selective pressure.

Using improved sequencing technologies, new subgroups of IncP-1 were discovered and the sequence-based information was used to design primer systems that targeted plasmids of the different subgroups (Bahl et al., 2009; Jechalke et al., 2013). PCR-Southern blot hybridization and real-time quantitative PCR (qPCR) allowed detecting and estimating IncP-1 plasmid abundance in total community DNA (TC-DNA) from various environmental settings (Götz et al., 1996; Heuer et al., 2012; Jechalke et al., 2013, 2014). Exogenous plasmid capturing was one efficient way to isolate these plasmids from environmental bacteria, without cultivation of their original host (Smalla et al., 2015). The plasmid capturing by biparental matings is based on the expression of accessory genes provided by the conjugative plasmid, including resistance(s) to antibiotics or heavy metals. The capturing by triparental matings involves a second donor carrying a small mobilizable plasmid and is based on plasmid mobilizing capacity (Smalla et al., 2015). Notably, IncP-1 plasmids were often obtained by both methods, and in triparental matings, IncQ/P-4 plasmids were used as mobilizable plasmids (Smalla et al., 2015). More than 40 IncP-1 plasmids previously sequenced have been exogenously captured; more than 30 were obtained by biparental mating, while only nine were captured by triparental mating (Supplementary Table S1).

Recently, we reported the increase of the relative abundance of IncP-1 plasmids in the rhizosphere of lettuce grown under field conditions in three non-polluted arable soils compared to bulk soil (Jechalke et al., 2014). DNA directly extracted from bulk soil or lettuce rhizosphere (TC-DNA) was analyzed by real time qPCR targeting the IncP-1 specific *korB* gene. The strongly increased relative abundance of *korB* copy numbers in the rhizosphere compared to bulk soil was rather unexpected and revealed significant gaps in our present understanding of the ecology and role of IncP-1 plasmids. In order to confirm the findings made with the rhizosphere bacterial communities of field-grown lettuce and to evaluate if the findings can be expanded to other crop plants, several greenhouse experiments were performed with lettuce (*Lactuca sativa* L.), tomato (*Solanum lycopersicum*), and potato (*Solanum tuberosum*) plants. TC-DNA was isolated from both the rhizosphere and the bulk soil, and the relative abundance of IncP-1 plasmids was determined by the ratio of *korB* copies and 16S rRNA (*rrn*) gene copies quantified in TC-DNA by qPCR. As a means to capture IncP-1 plasmids, we employed the triparental mating with a mobilizable IncQ/P-4 pSM1890 plasmid. IncP-1 plasmids were successfully captured in *Cupriavidus necator* from the rhizosphere bacterial communities of lettuce, tomato, and potato but not from bulk soil. A total of 14 IncP-1 plasmids were selected based on prior phenotypic and genotypic characterization. The insights gained from their long read sequencing are reported here.

## Materials and Methods

### Greenhouse Experiments, Sampling, and Sample Preparation

Lettuce [*Lactuca sativa* L.; cultivar (cv) Tizian], tomato (*Solanum lycopersicum*; cv Moneymaker), and potato (*Solanum tuberosum*; cv Arkola) were planted as seeds or tubers in diluvial sand [DS, arable soil whose characteristics were previously described (Rühlmann and Ruppel, 2005; Schreiter et al., 2014)], and grown under greenhouse conditions (16 h light, 20°C). Plants were watered with 2 g/L Wuxal^®^ Super (AGLUKON Spezialdünger GmbH & Co., KG, Düsseldorf, Germany), with an NPK ratio of 8-8-6 and micro-nutrients. In addition, pots filled with DS and kept unplanted under the same conditions were used as bulk soil controls (four replicates each). Ten (experiment I: lettuce) or six weeks (experiment II: tomato and potato) after transplanting, plants were destructively sampled (four replicate pots) and vigorously shaken to detach loosely bound soil. Greenhouse experiment III was performed with lettuce and tomato plants only to study the relative abundance of IncP-1 plasmids during different plant growth stages. Therefore, four plants sampled six, eight, and ten weeks after transplanting were analyzed.

Rhizosphere microbial fractions were obtained from the entire root system with the tightly adhering soil placed in Stomacher bags after homogenization with 15 mL of 0.85% NaCl using Stomacher 400 Circulator (Seward Ltd., Worthing, United Kingdom). The supernatants were collected, and the Stomacher step was repeated twice. The microbial pellet was obtained from the combined supernatants (45 mL) by centrifugation (16,000 × *g*, 10 min). The pellet was resuspended in 2 mL 0.85% NaCl, and 1 mL cell suspension was used for the exogenous plasmid isolation experiments. Five-gram bulk soil samples from unplanted pots were processed accordingly. The microbial pellet was harvested by centrifugation.

### Determination of the Relative Abundance of *korB* in TC-DNA

TC-DNA was extracted and purified from 500 mg of the rhizosphere or bulk soil-derived microbial pellets (four replicates each) as previously described (Jechalke et al., 2013; Blau et al., 2019). Total bacterial 16S rRNA gene (*rrn*) copy number was determined for the TC-DNA obtained from rhizosphere samples in addition to bulk soil as described in Suzuki et al. (2000). The abundance of *korB* gene, specific for IncP-1 plasmids, was quantified by TaqMan^TM^-based real-time PCR methods (Jechalke et al., 2013). The relative abundance of IncP-1 plasmids was calculated as ratio of *korB* gene copy numbers and the *rrn* copies of the corresponding samples and presented as Log_10_ values. The CFX 96 Real-time detection system (Bio-Rad Laboratories, Hercules, CA, United States) was used with primer sets, TaqMan^TM^ probes, and reference plasmids as previously described (Heuer et al., 2012; Jechalke et al., 2013). The statistical significance was tested by Tukey’s HSD test.

### Triparental Exogenous Plasmid Isolation

Triparental matings were performed with rifampicin (Rif) resistant *Cupriavidus necator* JMP228 as a recipient (which has been successfully used in different experiments as a recipient for capturing IncP-1 plasmids, reviewed in Smalla et al., 2015), *Escherichia coli* J53 (pSM1890) with the mobilizable IncQ/P-4 plasmid pSM1890 as a second donor (Tietze et al., 1989; Haagensen et al., 2002), and detached cells from rhizosphere and bulk soil samples as donors of mobilizing plasmids as previously described (Smalla et al., 2006). Plasmid pSM1890 encoded GFP and conferred streptomycin (Sm) and gentamicin (Gm) resistances. Overnight cultures of second donor and recipient strains were prepared with lysogeny broth (LB; Roth, Karlsruhe, Germany) supplemented with appropriate antibiotics. The detached cells were activated in 1:10 tryptic soy broth (TSB) medium (Becton Dickinson and Company, Sparks, MD, United States) and gently shaken (150 rpm, 2 h) at room temperature. Recipient, donor, and second donor cells were washed, harvested, and resuspended in 1:10 TSB. A mixture of cells was pipetted onto a Millipore filter (0.22 μm) placed on plate count agar (PCA) (Merck Corp., Kenilworth, NJ, United States) supplemented with cycloheximide (Cyc). After 24 h filter mating at 28°C, cells resuspended from the filters in 1 mL NaCl 0.85% and serial dilutions were plated on PCA with Cyc (200 μg/mL), Rif (50 μg/mL), Km (50 μg/mL), Gm (20 μg/mL), and Sm (50 μg/mL), and incubated for 48 h.

### Confirmation of the Transconjugants’ and Plasmids’ Identity and IncP-1 Plasmids Detection and Identification

Genomic DNA was extracted from each overnight culture of potential transconjugants from the lettuce (pTL), tomato (pTT), and potato (pTK) rhizosphere and recipient using Qiagen genomic DNA extraction kit (Qiagen, Hilden, Germany). The presence of *oriV* region (IncQ/P-4) of pSM1890 was confirmed by PCR with IncQ/P-4 specific primers. BOX-PCR performed as described by Rademaker and De Bruijn (1997) to confirm that the patterns were identical between those of transconjugants and the corresponding recipient. Plasmid DNA extraction from the pSM1890-positive transconjugants was performed as described previously (Smalla et al., 2000). Real-time qPCR was employed for screening the plasmid DNA for *korB* gene (Heuer et al., 2012; Jechalke et al., 2013). To confirm the presence of IncP-1 plasmids as well as to evaluate their diversity, the *korB* positive transconjugants were digested with *Not*I (Thermo Fisher Scientific). The digested plasmid DNA was subjected to 1% agarose gel (w/v) electrophoresis and then Southern-blot analysis was performed with IncP-1 mixed probes (targeting the *trfA* gene of α, β, ϵ, 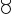, and δ subgroups) (Bahl et al., 2009; Wolters et al., 2014). The obtained restriction patterns were compared using GELCOMPAR II version 6.5 (Applied Math, Sint-Martens-Latem, Belgium). The clusters were constructed according to their similarity using Pearson’s correlation coefficients and hierarchical cluster method UPGMA (unweighted pair group method average algorithm means). Based on the restriction pattern comparisons, one representative plasmid was selected for sequencing.

### Whole Nucleotide Sequencing of Plasmids and Analyses

Plasmid DNA was extracted from the transconjugants using QIAGEN Plasmid Midi Kit (QIAGEN). SMRTbell^TM^ template library was prepared according to the instructions from Pacific Biosciences, Menlo Park, CA, United States. For preparation of 10 kb libraries 1 to 4 μg of each plasmid DNA was sheared using g-tubes^TM^ (Covaris, Woburn, MA, United States) and DNA was end-repaired and ligated overnight to barcoded SMRTbell^TM^ adapters applying components from the DNA/Polymerase Binding Kit P6 (Pacific Biosciences). Five to eight SMRTbell^TM^ templates were combined equimolar. Samples were either exonuclease-treated for removal of incompletely formed reaction products or subjected to a BluePippin^TM^ Size-Selection (Sage Science, Beverly, MA, United States) for DNA fragments greater than 4 kb. SMRT sequencing was carried out on the PacBio RSII (Pacific Biosciences) taking one 240-min movie for each SMRT cell. Assemblies of the multiplexed plasmid pools have been performed using the HGAP3 Whitelisting protocol within SMRTPipe 2.3.0 applying a genome size of 100 kb and a minimum subread length of 1 kb after demultiplexing using the RS_Subreads.1 protocol contained within SMRT Portal 2.3.0. Whenever possible, plasmids were circularized removing artificial redundancies at the ends of the contigs. Draft annotation was performed using Prokka (Seemann, 2014). The conserved genes in the IncP-1 plasmids were reannotated and named based on those in R751 (Thorsted et al., 1998), except for *kfrB* (upf54.8 in R751) and *kfrC* (upf54.4 in R751). Accessory genes including putative metabolic genes and/or transporter genes were subjected to BLAST^1^ to find similar sequences. Genotypic screening of antibiotic resistance genes in these plasmids was performed by using the comprehensive antibiotic resistance database (CARD) (Alcock et al., 2020). Comparative analyses of the plasmids were performed and visualized by Easyfig ver. 2.2.2 (Sullivan et al., 2011). The nucleotide sequences of genes encoding replication initiation protein (*trfA*) and relaxase (*traI*) of 130 selected IncP-1 plasmids (Supplementary Table S3) were aligned using ClustalW (Thompson et al., 1994) and the maximum likelihood method was used for the unrooted trees using MEGA 7 (Kumar et al., 2016). Visualization of plasmid maps was performed using SnapGene^2^. Plasmid sequences were deposited in GenBank under Accession Numbers MH392232 to MH392246.

## Results

### Influence of Plant Species and Age on the Relative Abundance of IncP-1 Plasmids

**FIGURE 1:**
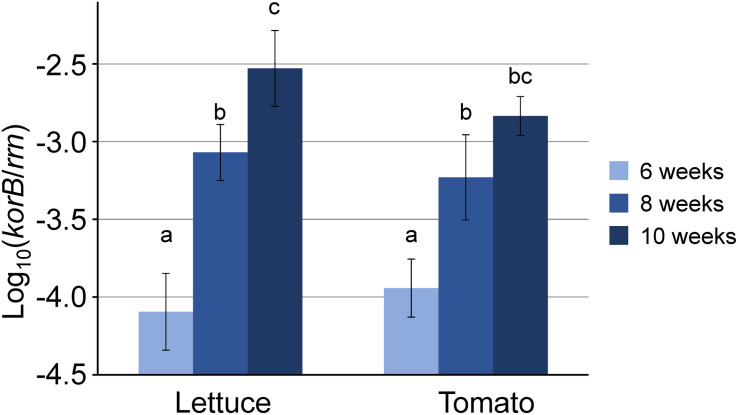
Relative abundance of IncP-1 plasmids log_10_(*korB*/*rrn*) in total community DNA (TC-DNA) from the rhizosphere of lettuce and tomato plants taken at different time points of plant development. a, b, and c indicate the significant differences (Tukey’s HSD test, *p* < 0.05, *n* = 4). bc indicates that significant differences were detected between the results of the rhizosphere of lettuce and tomato plants at 6 weeks and that of tomato at 10 weeks, but not between those of lettuce and tomato at 10 weeks.

The relative abundance of IncP-1 plasmids (ratio of *korB* to *rrn*) quantified by qPCR in TC-DNA showed an approximately one to two orders of magnitude increased relative abundance in the rhizosphere of lettuce and tomato plants compared to bulk soil (*t*-test, *n* = 4, *p* < 0.01 or Tukey’s HSD test, *n* = 4, *p* < 0.05), while this was not significant in the potato rhizosphere (Tukey’s HSD test, *n* = 4, *p* > 0.05, experiments I and II, Supplementary Figure S1). In addition, *korB* quantification in TC-DNA from the rhizosphere samples of lettuce and tomato plants collected at six, eight, or ten weeks after transplanting (greenhouse experiment III) revealed that for both plant species the relative abundance of IncP-1 plasmids significantly increased with plant age (Tukey’s HSD test, *n* = 4, *p* < 0.05, Figure 1).

### Exogenous Isolation of IncP-1 Plasmids Based on Their Mobilizing Capacity

From the rhizosphere of lettuce, tomato, and potato, 16, 51, and one IncP-1 plasmids were obtained, respectively, but none was obtained from bulk soil samples. Based on NotI restriction pattern analysis, 14 IncP-1 plasmids were selected, and their complete sequences were obtained. All plasmids captured from the lettuce rhizosphere had a unique restriction pattern, except for pTL50 that was representative of four transconjugants (Supplementary Table S2). In contrast, the restriction patterns of plasmids from the tomato rhizosphere fell into one of a number of groups, i.e., pTT11 was a representative of 21 plasmids, pTT47 of 12, pTT5 of three, and pTT60 of two (Supplementary Table S2). Phenotypic and genotypic screening of transconjugants did not indicate acquired antibiotic resistances.

### PacBio Sequence Analysis Revealed That IncP-1 Plasmids From the Rhizosphere Belong to Different Subgroups

**FIGURE 2:**
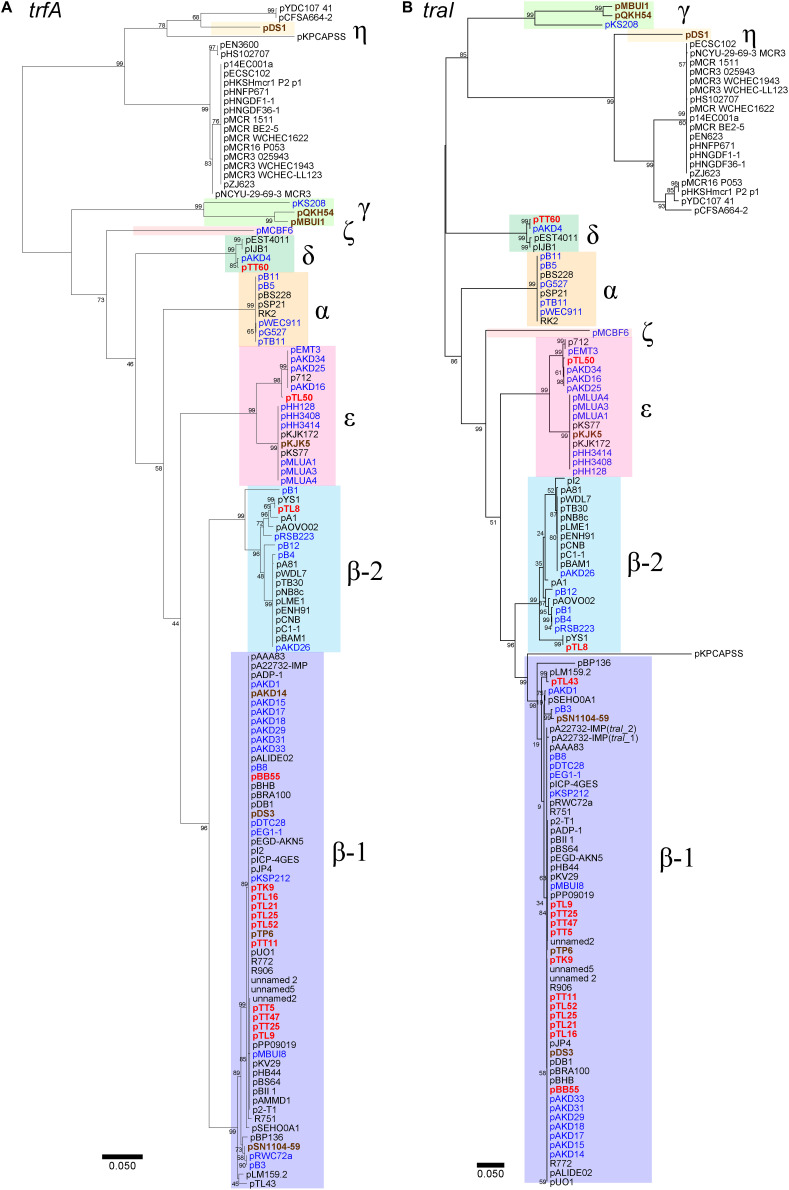
Phylogenetic analysis based on nucleotide sequences of **(A)** *trfA* (encoding replication initiation protein) and **(B)** *traI* (encoding relaxase) of IncP-1 plasmids. Plasmids with colors indicate that they were obtained by exogenous plasmid capturing, red for the obtained plasmids in the present study, blue for biparental mating, and brown for triparental mating. The accession numbers of other IncP-1 plasmids are listed in Supplementary Table S3. The unrooted trees were inferred by the neighbor joining method using Geneious Prime 2019. Bootstrap values are indicated at each node.

The sizes of 14 representative IncP-1 plasmids captured from the rhizosphere of lettuce (pTL8, pTL9, pTL25, pTL16, pTL21, pTL43, pTL50, and pTL52), tomato (pTT5, pTT11, pTT25, pTT47, and pTT60), and potato (pTK9) remarkably varied. Their sizes ranged from 39,671 bp to 107,234 bp for pTL50 and pTL9 plasmids, respectively (Supplementary Table S2). The phylogenetic analysis of the plasmids was performed based on the nucleotide sequence of *trfA* and *traI* genes encoding replication initiation protein and relaxase, respectively (Figure 2). The majority of the plasmids from all three rhizospheres (12 out of 14 plasmids) belonged to the IncP-1β subgroup (Figure 2 and Supplementary Table S2). Eleven IncP-1β plasmids were classified as IncP-1β1 subgroup and the pTL8 plasmid belonged to the IncP-1β2 subgroup. Plasmid pTT60 showed >99% nucleotide sequence identity of *trfA* and *traI* with the new IncP-1δ group prototype plasmid pAKD4. The only plasmid in this study without any MGEs or accessory genes was the IncP-1ε plasmid pTL50. Eleven plasmids had type II toxin-antitoxin systems (TAs), which were encoded by *kluAB* genes in their backbone regions (Supplementary Figure S2). Six of them (pTK9, pTT11, pTL16, pTL21, pTL25, and pTL52) had a putative RelEB system encoding riboendonuclease as toxin that cleaves mRNA on translating ribosomes (Christensen et al., 2001). The other five (pTL9, pTL43, pTT5, pTT25, and pTT47) had a putative ParED system encoding inhibitor of DNA gyrase as toxin (Jiang et al., 2002) (Supplementary Figure S2). No previously known antibiotic resistance genes were identified in any captured plasmids by searching the comprehensive antibiotic resistance database (CARD).

#### The Diversity of IncP-1β Subgroup Driven by MGE

**FIGURE 3:**
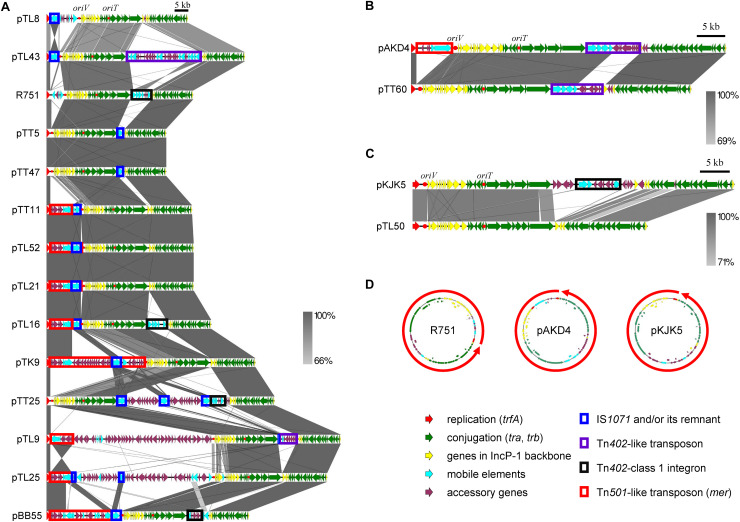
Comparisons of the whole genetic structure for the captured plasmids. **(A)** IncP-1β with representative plasmids R751 and pBB55 from manure soil, **(B)** IncP-1δ with pAKD4, and **(C)** IncP-1ε with pKJK5. *trfA* was set as the right end of the linear plasmid maps, and the start points and directions of the representative plasmids in the maps are shown by red arrows in panel **(D)**. Block arrows with colors indicate coding sequences (CDSs) and their predicted functions (red for replication, green for conjugation, yellow for other genes in IncP-1 backbone, light blue for genes related to mobile genetic element, and magenta for accessory genes). Homologous regions are indicated by frame areas. The key mobile genetic elements (IS*1071*, Tn*402*, Tn*402*-integron, and Tn*501*) are shown by colored rectangular shapes that are shown below the figure.

The comparison of the sequences beyond the backbone genes revealed that the diversity of the set of plasmids studied here was driven by MGEs and accessory genes (Figure 3 and Supplementary Table S2). The two smallest IncP-1β1 plasmids originating both from the tomato rhizosphere, pTT5 and pTT47 (43,580 and 43,582 bp), are likely identical. Interestingly, they consisted only of the plasmid backbone genes except for the presence of IS*1071* linked to a gene coding for a hypothetical protein. Furthermore, sequence analysis showed that the parA gene was lost from both plasmids, likely due to the insertion of IS*1071* at this site (Supplementary Figure S2).

#### Tn*501*-Like Transposon

**FIGURE 4:**
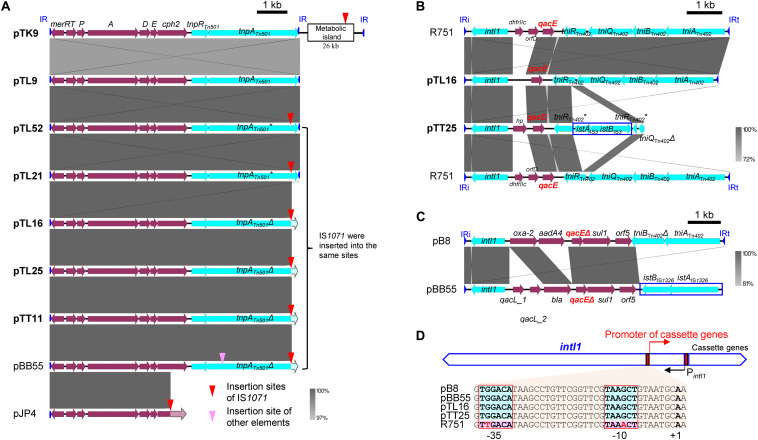
Comparisons of the genetic structure for the key mobile genetic elements (MGEs), Tn*501* **(A)** and Tn*402*-class 1 integrons **(B,C)**. CDSs and homologous regions are indicated by block arrows and framed areas, respectively. Inverted repeats (IRs) are shown by blue triangles. * or Δ indicates that the gene was disrupted or truncated by other MGEs, respectively. **(A)** Comparisons of Tn*501* containing the *mer* operon found in IncP-1β-1 plasmids with pJP4, which has a *mer* operon. The insertion sites of IS*1071* are pointed by red triangles. pTK9 had another inverted repeat of Tn*501* in downstream of the metabolic island with IS*1071* insertion. pBB55 had another insertion in the *tnpA*_Tn_*_501_* gene shown by pink triangles. **(B)** Comparisons of Tn*402*-class 1 integron like regions in pTL16 and pTT25 with that in R751. IS*3*-family element is shown as a rectangle. **(C)** Comparisons of Tn*402*-class 1 integron-like regions in pBB55 (captured from manure soil) with those in pB8. IS*1326*-family element is shown by a rectangle. **(D)** Comparisons of promoters for gene cassettes in the Tn*402*-class 1 integrons. Red boxes show –35 and –10 region of the gene cassette and +1 indicates transcription start point. The boxes with light blue indicate PcW promoter (pB8, pBB55, pTL25, and pTT25), while those with pink indicate PcS promoter in R751.

The Tn*501*-like transposon linked to a *mer* operon was detected on seven of the 11 IncP-1β1 plasmids studied here (Figure 3A) and the transconjugants harboring these plasmids indeed grew on a mercury chloride-containing medium (data not shown). In all plasmids with Tn*501* the insertion site was between *trfA* and *oriV.* However, a closer sequence comparison revealed a few distinct differences that were caused mainly by the insertion site of IS*1071* and truncation of the Tn*501 tnpA*_Tn_*_501_* coding for the transposase (Figure 4A and Supplementary Text).

#### Tn*402*/Class 1 Integrons

Complete or remnants of Tn*402*-like transposons were found in the four IncP-1β1 plasmids, pTL9, pTL16, pTL43, pTT25, and in the IncP-1δ plasmid pTT60 (Figure 3A and Supplementary Figure S3). Despite the differences in the backbone of the two plasmid subgroups, the integration site was always between *traC* and *parA*. In pTL16 and pTT25, the complete or the remnant of the Tn*402*-like transposon was linked to a class 1 integron, which was highly similar to the constellation in the prototype plasmid R751 (Figure 4B). In contrast to R751, the class 1 integrons on pTL16 and pTT25 did not carry any gene cassettes conferring antibiotic resistances but an intact *qacE* gene instead (Figure 4B). In R751, the class 1 integron is linked to a *qacE* in addition to an antibiotic resistance gene cassette (*dhfrII*, Figure 4B). Notably, nucleotide sequences of the promoters of gene cassettes on the integrons in pTL16, pTT25, and R751 were different; as shown in Figure 4D, the former two were identical with PcW promoters, while that in R751 was identical with PcS promoter (Labbate et al., 2008; Chamosa et al., 2017; Ghaly et al., 2017).

#### IS Elements

Different IS elements were identified in this set of IncP-1 plasmids, but by far the most frequently detected IS element was IS*1071* (Figure 3A and Supplementary Figure S3). This IS element, or its remnant, was detected in all IncP-1β plasmids, and in eight plasmids, IS*1071* was integrated between *trfA* and *oriV* (Figure 3A and Supplementary Figure S3). In the remaining plasmids, it was inserted between *traC* and *parA* (Figure 3A and Supplementary Figure S3). In seven plasmids, a complete IS*1071* was detected, and in three plasmids, the IS*1071* also contained direct repeats (Supplementary Table S2). In pTT11 a Δ*tnpA*_IS_*_1071_* frame shift was detected in the IS*1071* integrated into the Tn*501* (Supplementary Table S2).

#### Metabolic Genes of IncP-1β Plasmids

Five IncP-1β plasmids captured from potato or lettuce rhizosphere carried putative metabolic gene clusters or even complete operons and therefore were larger than most of the remaining plasmids. In pTK9, the only plasmid captured from potato rhizosphere, a complete set for catechol degradation genes also related to chloroaniline degradation was inserted between *trfA* and *oriV* (Figure 3A and Supplementary Figure S3). The metabolic genes were localized downstream of the *mer* operon/Tn*501* followed by IS*1071* (Figure 3A and Supplementary Figure S3). Interestingly, another Tn*501* inverted repeat was found downstream of IS*1071* (Supplementary Figure S3). The IncP-1β2 plasmid pTL8 carried a chlorite dismutase gene inserted between *trfA-oriV* in a Tn*3*-like transposon downstream from a complete IS*1071* with direct repeat (Supplementary Figure S3). Plasmid pTL9 carries a remarkably large fragment (50 kb) between *trfA* and *oriV*, showing high identity (>99%) with the genomic DNA of *Cupriavidus basilensis* strains X1 and 4G11 (Supplementary Figure S3). This region contained putative genes for glycolate and pyruvate metabolisms (50 kp). In addition, this plasmid carried putative molybdenum transporter genes *modEAGC* inserted between *traC* and *parA* linked to Tn*402* (Supplementary Figure S3). Putative novel dioxygenases (Rieske type) and C4-organic transporters were identified in pTL25 downstream of the *mer* operon/Tn5*01* and two remnant IS*1071* copies (Supplementary Figure S3). In pTL43, the insertion of extradiol dioxygenase genes into a complete Tn*402* was observed between *tra*C and *parA* (Supplementary Figure S3). The metabolic genes were linked to three IS*21*, two of which were identical (Supplementary Figure S3).

A very curious observation was that a large proportion of plasmid pTL21 seemed to have lost a large metabolic island. pTL21 had been considered to carry putative polyaromatic hydrocarbon (PAH) ring hydroxylating dioxygenase gene, because a PCR product was detected with primers for PAH-*rhd*α (Ding et al., 2010). In-depth analyses in the assembly process of pTL21 revealed that only a smaller proportion of pTL21 carried the above gene as a part of *nag* operon, named as pTL21^∗^(Supplementary Text). pTL21^∗^ was 81,088 bp in size and carried a complete *nag* operon, which was integrated into IS*1071* linked to Tn*501-mer* operon (Supplementary Text and Supplementary Figure S4). The putative naphthalene degradative (*nag*) genes were flanked by the two copies of IS*1071* in pTL21^∗^, which might have been lost by homologous recombination between them (Supplementary Figure S4). This was a possible reason that the majorly assembled plasmid (i.e., pTL21) did not carry the *nagAg* operon.

## Discussion

Quantification of IncP-1 plasmids in TC-DNA revealed that remarkably, these plasmids increased in relative abundance in the rhizosphere of lettuce and tomato plants along with the plant age. These results obtained under greenhouse conditions confirmed our previous data on the rhizosphere of lettuce grown under field conditions in three different soils (Jechalke et al., 2014). However, the increase in relative abundance in the rhizosphere of lettuce compared to bulk soil was stronger under field conditions (more than two orders of magnitude) compared to the pot experiment. These plant species-dependent differences were likely due to the differences in amount of root exudates and deposits (Neumann et al., 2014) shaping the plant species-dependent bacterial community. Although we did not perform amplicon sequencing of 16S rRNA genes amplified from rhizosphere TC-DNA of lettuce, tomato, and potato in the present project, this was done for lettuce and potato both grown under field conditions in DS soil. The rhizosphere microbiome was distinct (unpublished) likely due to differences in exudate composition.

The exogenous plasmid capturing methods have a limitation in so far as the original host(s) of the obtained plasmids is not determined (Smalla et al., 2015). Several potential hosts of IncP-1 plasmids including *Pseudomonas*, *Variovorax*, and *Burkholderia* were previously reported as rhizosphere responders (genera with a significant increase in relative abundance in the rhizosphere compared to bulk soil) of lettuce grown in DS under field conditions (Schreiter et al., 2014). Currently, except for one isolate, the IncP-1 plasmids were not successfully detected by PCR of bacterial isolates obtained from the rhizosphere of lettuce and tomato (data not shown). This was probably because the original hosts of IncP-1 plasmids were not cultivable or because they did not belong to the dominant bacterial isolates. To identify the original hosts of the obtained plasmids, further in-depth analysis with HiC method as reported in Stalder et al. (2019) will be necessary.

In sewage sludge or manure, high concentrations of pollutants likely provide a selective advantage of carrying IncP-1 plasmids because they typically carried multiple antibiotic resistance genes (Schlüter et al., 2007). In contrast, the rhizosphere samples studied here originated from plants grown in unpolluted soil.

Most unexpected was the high diversity of IncP-1 plasmids captured. In contrast, only one dominant IncP-1 plasmid type was captured from a soil treated with manure spiked with doxycycline (Blau et al., 2019) by biparental exogenous isolation as previously described (Blau et al., 2018; Supplementary Text). Forty plasmids captured from this soil (from three soil replicates) displayed identical restriction patterns (data not shown). One representative of these plasmids, pBB55, was sequenced in the same PacBio sequencing run as the 14 IncP-1 plasmids from the plant rhizosphere and represented a multi-resistance plasmid carrying a *tet*(C) gene conferring resistance to doxycycline but also others such as macrolide resistance genes *msr*(E) and *mph*(E), *mer* genes conferring resistance to mercury, aminoglycoside resistance genes *aph(3″)-Ib* and *aph(6)-Id*, beta lactamase gene *blaOXA-2*, and *emrE* multidrug efflux protein genes (Supplementary Figure S5, GenBank accession no. MH392232). These genes were contained by several transposons and integrons, and they were inserted into the two insertion hot spots (Supplementary Figure S5) like the other obtained IncP-1β plasmids (Supplementary Figure S3). One of them was between *trfA* and *oriV*, in which *tet*(C), *msr*(E), and *mph*(E) genes were contained by a composite transposon of IS*26*s, *aph(3″)-Ib* and *aph(6)-Id* were contained by a Tn*3*-like transposon, and *mer* genes were contained by Tn*501* remnant with IS*1071* (Supplementary Figure S5). The other was between *parA* and upf30.5, in which *emrE* was in Tn*402*-class 1 integron remnant (with IS*1326*) in addition with sulfonamide resistance gene *sul1* and quaternary ammonium compound resistance gene *qacE*Δ (Supplementary Figure S5). We propose that plasmid diversity observed under strong selective conditions including high pollutants and/or antibiotic exposure is lower due to the increased relative abundance of host populations carrying IncP-1 plasmids that provide fitness advantages by their accessory genes. In contrast, the plasmids from the rhizosphere of plants grown in unpolluted soils showed higher diversity, and none of them carried previously known antibiotic resistance genes by searching in CARD. The presence of highly diverse IncP-1 plasmids suggested that the role of IncP-1 plasmids in unpolluted rhizosphere might be not only be conferring resistance to antibiotics, disinfectants, heavy metals, and/or degradation of xenobiotics (Hsu and Bartha, 1979; Top and Springael, 2003; Smets and Barkay, 2005) but also providing genetic flexibility of microbes in rhizosphere by a high rate of gene acquisition and loss via various IncP-1 plasmids.

The present study points to the important role of plasmid-localized MGEs in the acquisition of accessory genes and plasmid evolution.

Tn*501* was always linked to mercury resistance in seven of the sequenced IncP-1 plasmids (Figure 3A and Supplementary Figure S3). Lilley and Bailey (1997) reported that plasmids with mercury resistance genes were isolated from the rhizosphere of sugar beets grown in arable field soils. However, in their report, the mercury resistance operon was carried by large *Pseudomonas* plasmids (Lilley and Bailey, 1997). To date, we can only speculate about the widespread dissemination of mercury resistance genes, and mercury resistance might provide a fitness advantage to their hosts. The amount of mercury compounds in soil might be due to pedogenesis, their presence in organic fertilizers (sewage sludge, manure), or their usage as fungicide, e.g., for treating seeds.

The class 1 integrons were similarly localized on the IncP-1 plasmids from unpolluted rhizosphere as those present on the prototype IncP-1β1 plasmid, R751 found in clinical isolate (Jobanputra and Datta, 1974). Sequence comparison of the class 1 integron identified in pTL16 from the lettuce rhizosphere displayed a high similarity to that in R751, except that the latter plasmid contained two additional gene cassettes with the *dhfr* gene conferring the trimethoprim resistance that initially led to its isolation. The class 1 integron of both plasmids carried an intact *qacE* gene, instead of *qacE*Δ*1*, which is presently far more frequently distributed (Ghaly et al., 2017). Indeed, the integron in pBB55 carried the *qacE*Δ*1* gene (Figure 4C), which was also similarly found in another IncP-1β plasmid, pB8, obtained by biparental plasmid capturing from an activated sludge (Schlüter et al., 2005). pTL16 seems to carry an ancestral class 1 integron, although loss of the antibiotic resistance gene cassettes localized on R751 cannot be excluded. Ghaly et al. (2017) reported an *Enterobacter cloacae* isolated from baby spinach leaves that also carried a class 1 integron inserted into a Tn*402* localized on an IncP-1β plasmid, pOP-I. They argued that plants eaten raw might be an important transmission route and a likely link from the environment to humans. Further, the promoters of these integrons in pTL16, pTT25, and also in pBB55 and pB8 were identical to “weak” PcW promoters (Figure 4D), which are considered to be “ancestral” promoters conserved in chromosomal integrons of “environmental” strains, while that in R751 was “strong” PcS promoter, which is common in “clinical” integron cassette arrays and considered to be an “evolved” promoter (Labbate et al., 2008; Chamosa et al., 2017; Ghaly et al., 2017). These facts indicate that the IncP-1 plasmids obtained from the rhizosphere possessed relatively ancestral types of class 1 integrons.

In five plasmids, MGEs were linked to metabolic degradative genes. The in-depth exploration of the metabolic genes linked to the IncP-1 plasmid captured from the rhizosphere and their contribution to host fitness deserves further studies. The products of these genes might be advantageous for the survival of host(s) of these plasmids in rhizosphere environments. As for pTL21, here we can only speculate that during growth of transconjugant, the presence of the *nag* operon was a metabolic burden and disadvantageous under the growth conditions on nutrient media. This might well present a general strategy to lower the metabolic burden of the population. In the rhizosphere, the presence of the *nagAg* operon might not represent a disadvantage due to lower growth rates and the presence of aromatic compounds in the root exudates (Neumann et al., 2014; Windisch et al., 2017).

pTL50 assigned to the IncP-1ε subgroup could represent the first archetype of the IncP-1ε group only carrying core genes (Figure 3C). Recently, we isolated numerous IncP-1ε plasmids conferring sulfadiazine or tetracycline resistance from bacteria of manure, digestates of biogas plants, and manure-treated soils by exogenous plasmid capturing (Heuer et al., 2012; Wolters et al., 2015). Similar to pKJK5, originally isolated from manure-treated soils (Bahl et al., 2007), all these plasmids carried class 1 integrons with *tet*(A) gene and their diversity was proposed to be driven by different sets of other gene cassettes (Heuer et al., 2012; Jechalke et al., 2014; Wolters et al., 2015). In contrast, IncP-1ε plasmids originating from mercury or 2,4-D polluted soils including pAKD34, pAKD25, pAKD16, and pEMT3 did not contain class 1 integrons but *mer* genes or metabolic genes (Sen et al., 2011; Sen et al., 2013; Li et al., 2016). pTL50 can be an archetype plasmid of them as a vector for genes in these polluted environments.

## Conclusion

The discovery that bacterial populations carrying IncP-1 plasmids are increased in relative abundance in the rhizosphere of plants grown in unpolluted soil in a plant species-dependent manner is of importance as these self-transmissible plasmids might represent important shuttles of genes conferring adaptive traits or MGEs ready to recruit them. Here we showed that the exogenously captured IncP-1 plasmids exhibited an unexpectedly high diversity. As described in Brown et al. (2013), it is still unclear whether the IncP-1 plasmids with a few or no accessory genes are advantageous for their hosts. They might be advantageous because they allow rapid and dynamic acquisition or loss of accessory genes due to the presence of MGEs that were identical or highly similar to the ones found on the clinical IncP-1 plasmids conferring multiple antibiotic resistances.

## Data Availability Statement

The datasets presented in this study can be found in online repositories. The names of the repository/repositories and accession number(s) can be found in the article/Supplementary Material.

## Author Contributions

KS conceived, designed, and supervised the study. MS, EN, TE, IW, CS, BB, and JO performed the experiments and data analyses. MS, EN, KB, SJ, BB, JO, and KS wrote, reviewed, and edited the manuscript. All authors contributed to manuscript revision, read, and approved the submitted version.

## Conflict of Interest

The authors declare that the research was conducted in the absence of any commercial or financial relationships that could be construed as a potential conflict of interest.

## Footnotes

1 https://blast.ncbi.nlm.nih.gov/Blast.cgi

2 http://www.snapgene.com/
