# Supplementary material for: Plant Species-Dependent Increased Abundance and Diversity of IncP-1 Plasmids in the Rhizosphere: New Insights Into Their Role and Ecology
Source: Front Microbiol. 2020 Nov 27;11:590776. doi: 10.3389/fmicb.2020.590776 (PMC7728920; doi:10.3389/fmicb.2020.590776)
Supplement: Supplementary file 1 [file Data_Sheet_1.pdf]

Supplementary Material

Plant species-dependent increased abundance and diversity of IncP-1 plasmids in the rhizosphere – new insights into their role and ecology

Masaki Shintani, Eman Nour, Tarek Elsayed, Khald Blau, Inessa Wall, Sven Jechalke, Cathrin Spröer, Boyke Bunk, Jörg Overmann Kornelia Smalla

Corresponding author:

Professor Dr. Kornelia Smalla kornelia.smalla@julius-kuehn.de

The Supplementary information includes

- ✓ Supplemental text
- ✓ Figures S1-S5
- ✓ Tables S1-S3

Supplemental text

Closer sequence comparison of Tn*501* and insertion sites of IS*1071*

In two of the plasmids, pTK9 and pTL9, Tn*501* seems to be intact while in pTL52 and pTL21, the IS*1071* was inserted into the *tnpA*Tn*501* gene (labeled as* in Fig. 4A). In three plasmids, pTL16, pTL25, and pTT11, the *tnpA*Tn*501* gene was truncated with IS*1071*, and notably, the insertion sites of IS*1071* were identical in those plasmids (Fig. 4A). Three of the Tn*501* carrying IncP-1β1 plasmids, pTT11, pTL21 and pTL52 seemed to be similar in size (53,011-53,601 bp). They all carried a Tn*501* linked to a complete *mer* operon and the differences among these plasmids were mainly due to the frame shift of *tnpA*IS*1071* and to the truncation of *tnpA*Tn*501* (Fig. 4A, Fig. S3). In pTL52 and in pTL21, the *tnpA*Tn*501* was complete but interrupted through the insertion of IS*1071*, while in pTT11 the IS*1071* was inserted at the end of the truncated *tnpA*Tn*501* (Fig. 4A). Exactly this was also observed for the sequence of pTL16 (Fig. 4A), which is larger in size (59,989 bp) due to the carriage of an additional MGE (Fig. 3A, Fig. S3). Similarly, the IS*1071* was inserted into the 3′ end of the truncated *tnpA*Tn*501* in pTL25 (102,276 bp) (Fig. 4A), which carried additional MGEs (Fig. 3A, Fig. S3). Both plasmids with an intact- and likely-active Tn*501* contained accessory metabolic genes either between *trfA* and *oriV*, which was downstream of *tnpA*Tn*501* linked to IS*1071* (pTK9) or between *traC* and *parA* (pTL9) (Fig. 3A, Fig. S3).

Exogenous isolation of plasmid pBB55 by biparental mating

Capturing tetracycline resistance-conferring conjugative plasmids was attempted from sandy soil and lettuce rhizosphere that were untreated, manure-treated, and treated with manure supplemented with doxycycline (50 and 100 mg/kg soil dry weight). Bacterial fractions of bulk soil and lettuce rhizosphere were prepared as described in the study of Blau et al. (2019). The filter mating assay using *Pseudomonas putida* KT2442 (*gfp*-tagged, kanamycin and rifampicin resistant) was done according to Wolters et al. (2015). Tetracycline resistant transconjugants were only captured from bulk soil amended with manure containing doxycycline (50 mg/kg soil dry weight). The transfer frequency of tetracycline resistant transconjugants was 1.59×10^{-6} (ratio colony forming units of transconjugants and recipient). Forty transconjugants were picked for further analysis. All transconjugants carried identical plasmids as revealed by restriction digestion analysis and one, plasmid pBB55, was selected for sequencing.

Assembly of pTL21 and pTL21*

Eight contigs (716 - 116,849 bp) were found for the first assembly of pTL21, and six of them showed 99-100% identities with the chromosomal DNA of *Ralstonia eutropha* JMP134, which is a closely related strain of the recipient, *Cupriavidus necator* JMP228. One of the other two contained *gfp* and streptomycin and gentamycin resistance genes indicating that it was a fragment of pSM1890, a mobilizable plasmid. The last one contig (117 kb) carried two IncP-1 plasmid backbone regions involved in plasmid replication, maintenance and conjugative transfer, two *mer* genes. Putative naphthalene degradative genes, *nagR*, *nagABCDEFGHIJKLM*, and *nagQ*, were also found, which were flanked by two copies of IS*1071* on it. Because the DNA region containing one of *mer* genes

and the *nag* gene cluster showed much lower read coverage in the contig, two contigs were reassembled with raw reads data. In conclusion, there were two kinds of plasmids, i.e., *nag* gene-containing plasmid (pTL21*, 81,088 bp) and *nag* gene-free plasmid (pTL21, 53,599 bp), while both shared the same plasmid backbones (Figure S4).

References

Blau, K., Jacquiod, S., Sørensen, S. J., Su, J.-Q., Zhu, Y.-G., Smalla, K., et al. (2019). Manure and doxycycline affect the bacterial community and its resistome in lettuce rhizosphere and bulk soil. *Front Microbiol* 10, 725.

Wolters, B., Kyselková, M., Krögerrecklenfort, E., Kreuzig, R., and Smalla, K. (2015). Transferable antibiotic resistance plasmids from biogas plant digestates often belong to the IncP-1ε subgroup. *Front Microbiol* 5, 765.

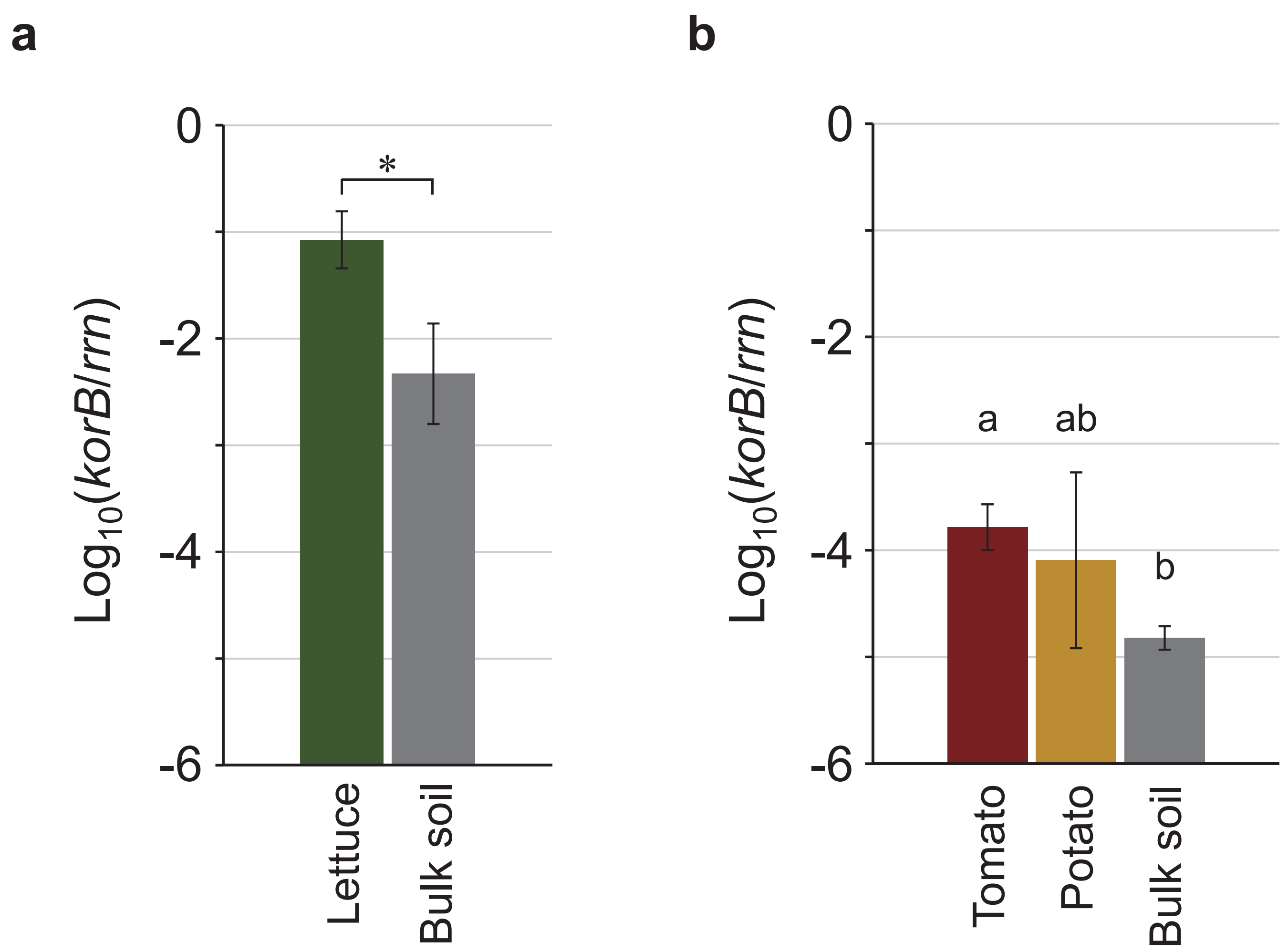

Fig. S1. Relative abundance of IncP-1 plasmids $\log^{10}$(*korB*/*rrn*) in total community DNA (TC-DNA) from the rhizosphere of lettuce and bulk soil in Experiment I (**a**), and those of tomato, potato plants and bulk soils in Experiment II (**b**). '**' indicates the significant differences (*t* test, p< 0.01) in panel **a**. 'a' and 'b' indicate the significant differences (Tukey HSD test, $p < 0.05$, n = 4). 'ab' indicates that significant differences were detected between the results of tomato and bulk soil, but however, not between those of tomato and potato, or between those of potato and bulk soil in panel **b**.

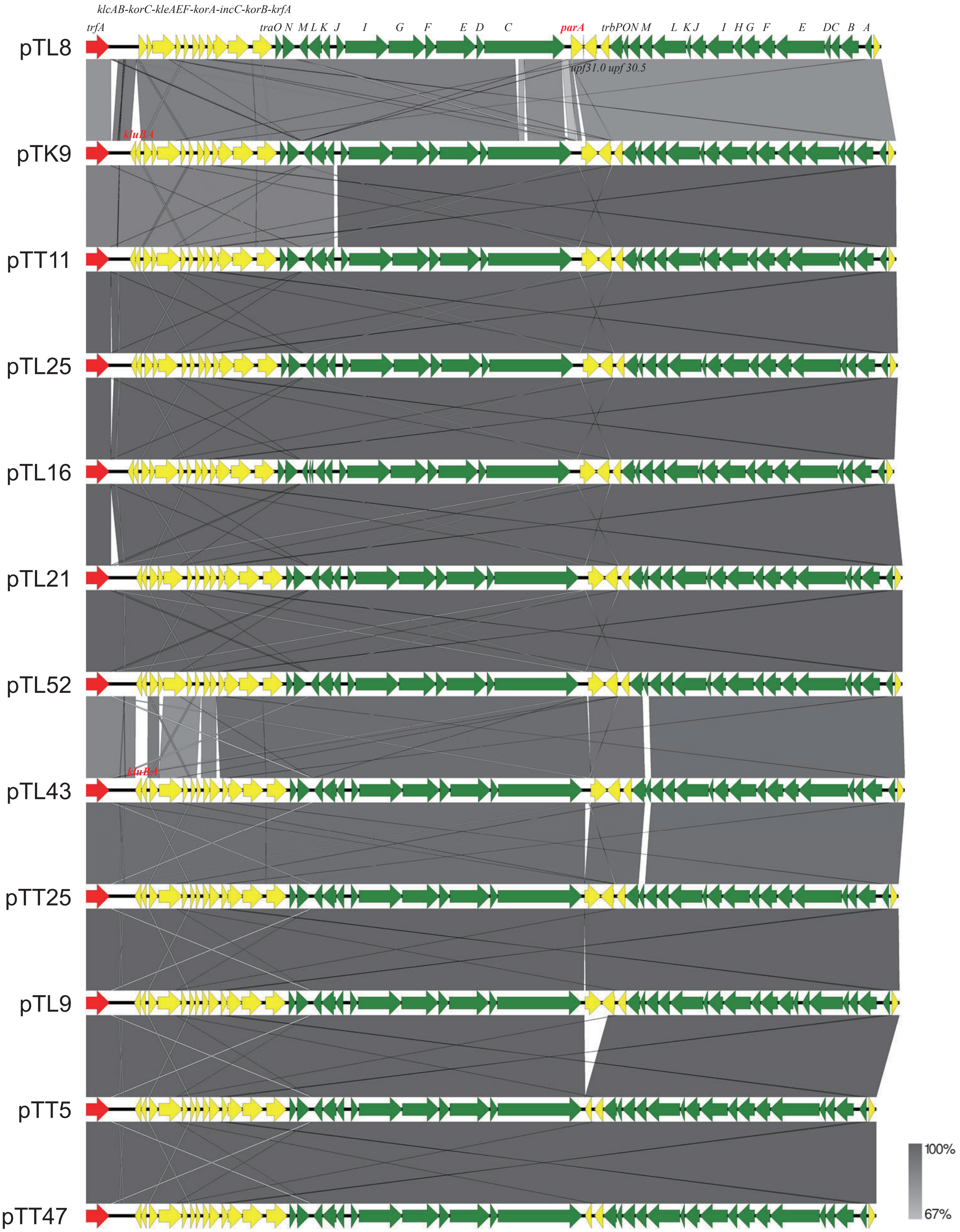

Fig. S2. Comparisons in the genetic structure of the IncP-1β backbones in the captured plasmids. The drawing way is identical with the Fig. 3 in main text, *trfA* was set as the right end of the linear plasmid maps. Block arrows indicate CDSs and the homologous regions are indicated by frame areas.

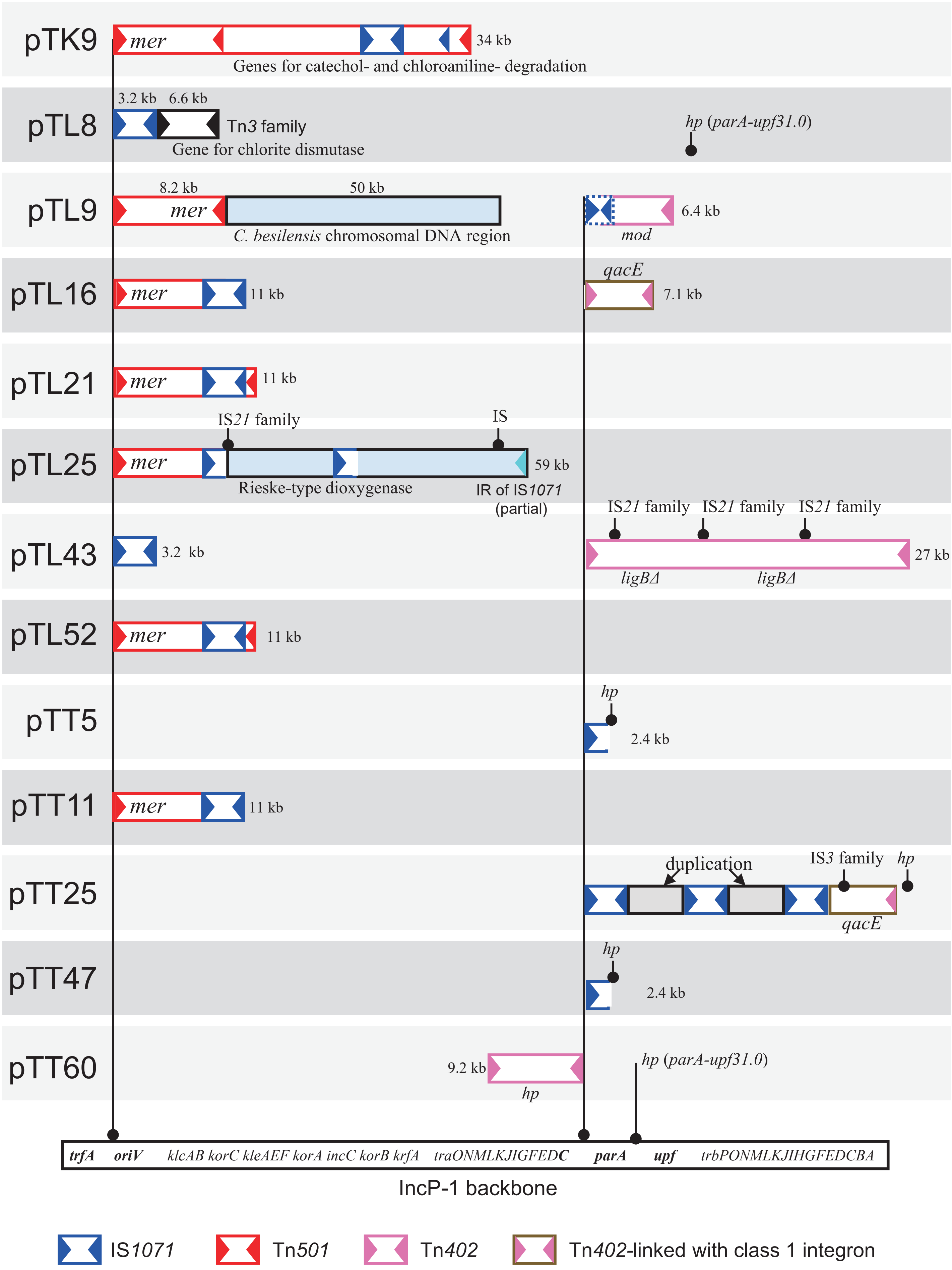

Fig. S3. Insertion sites of accessory genes including MGEs in the captured plasmids. Rectangles represent the inserted regions, MGEs are shown with their inverted repeats (triangles), in blue (IS*1071*), red (Tn*501*), pink (Tn*402*), and brown (Tn*402* with class 1 integron).

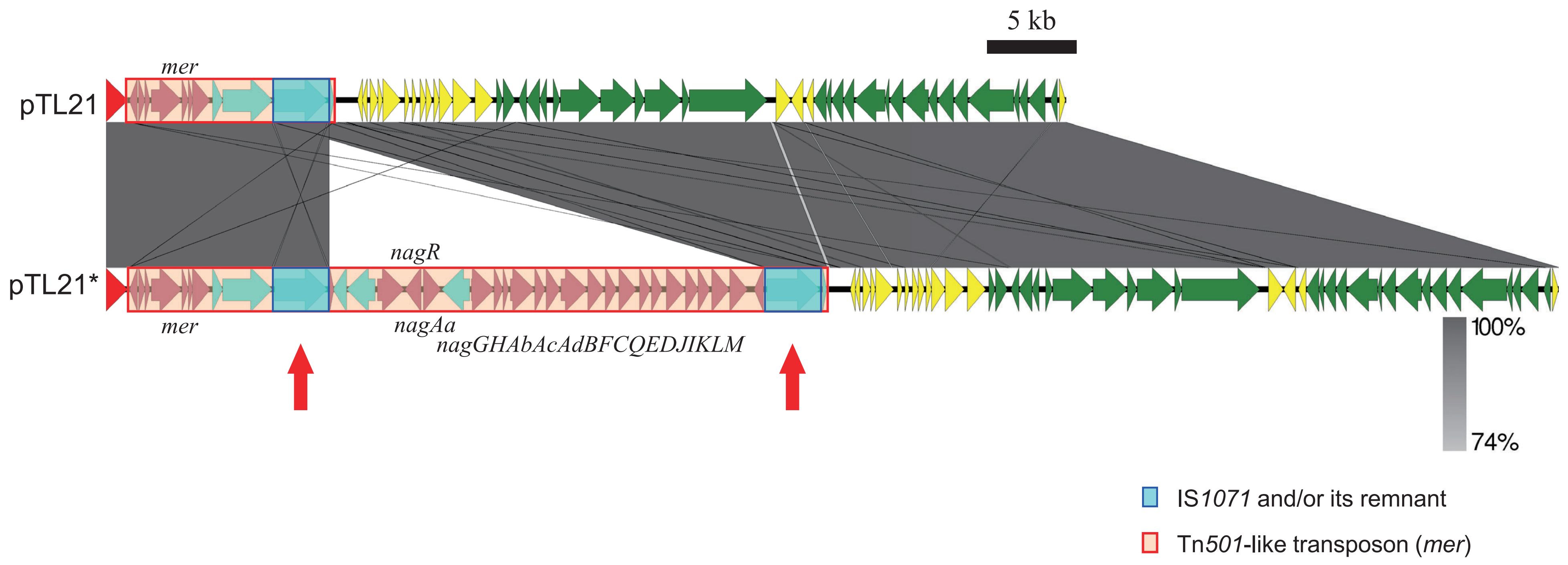

Fig. S4. Comparison of genetic structure between pTL21 and pTL21*. Putative naphthalene degradative (*nag*) operon might be lost from the majority of pTL21 by homologous recombination between the identical IS*1071* (red arrows). The drawing way is identical with the Fig. 3 in main text, *trfA* is set as the right end of the linear plasmid maps.

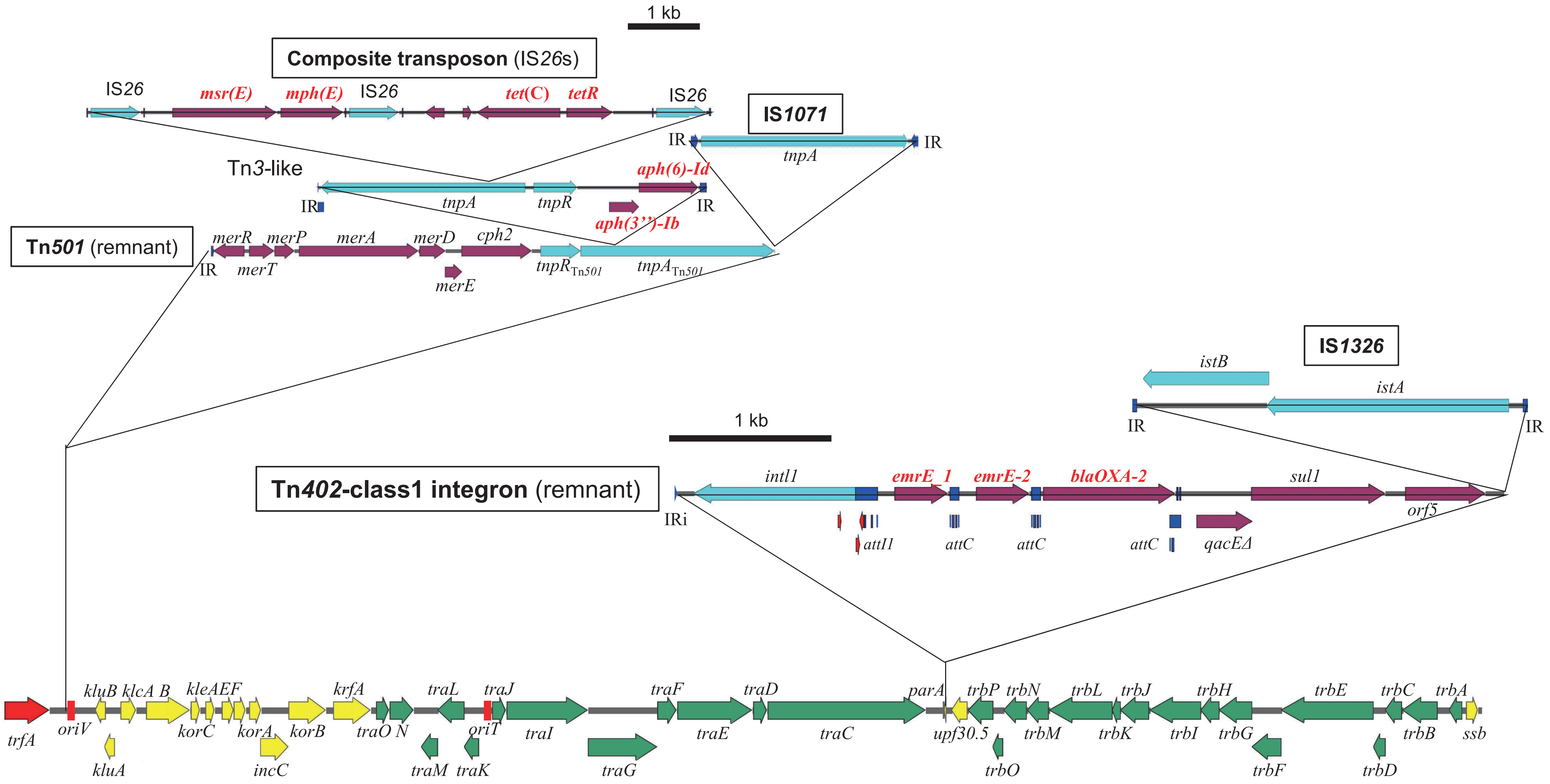

Fig. S5. Transposons and integron with antibiotic resistance genes inserted into pBB55. The drawing way is identical with the Fig. 3 in main text, *trfA* is set as the right end of the linear plasmid backbone. Names of antibiotic resistance genes are shown in red.

Table S1. Lists of IncP-1 plasmids obtained by exogenous plasmid capturing.

Plasmid name	Size (bp)	Subgroup of IncP-1 group	Accessory genes	Methods of exogenous plasmid capturing	Isolation source
pAKD1	58246	Beta-1	*mer, aadA*	Biparental	agricultural soil
pAKD14	48624	Beta-1	*mer*	Biparental/triparental	agricultural soil
pAKD15	66550	Beta-1	*mer* , ring-hydroxylating dioxygenase	Biparental	agricultural soil
pAKD16	74971	Epsilon	*mer* , alcohol dehydrogenase	Triparental(IncQ/P-4)	agricultural soil
pAKD17	51755	Beta-1	*mer*	Biparental	agricultural soil
pAKD18	67298	Beta-1	*mer*	Biparental	agricultural soil
pAKD25	75067	Epsilon	*mer* , *tfd* , gentisate dioxygenase, enoyl-CoA hydrase	Biparental	agricultural soil
pAKD26	82401	Beta-2	*mer* , *tfd* , gentisate dioxygenase, enoyl-CoA hydrase	Biparental	agricultural soil
pAKD29	55020	Beta-1	*mer*	Biparental	agricultural soil
pAKD31	54140	Beta-1	*mer*	Biparental	agricultural soil
pAKD33	57974	Beta-1	*mer* , ring-hydroxylating dioxygenase	Biparental	agricultural soil
pAKD34	86438	Epsilon	*mer* , dioxygenase for aromatic compounds	Biparental	agricultural soil
pAKD4	56803	Delta	*mer*	Biparental/triparental	soil
pB1	58290	Beta-2	*tetA*	Biparental	municipal waste water treatment plant
pB11	66911	Alpha	*mer*	Biparental	activated sludge
pB12	64393	Beta-2	*tetAR* , *qacE* delta1, *mph*	Biparental	municipal waste water treatment plant
pB3	56167	Beta-1	*aadA2* , *tet*	Biparental	activated sludge
pB4	79370	Beta-2	*str* , *mexCDoprJ*	Biparental	activated sludge
pB5	64696	Alpha	*aacC1* , *aacA4*	Biparental	activated sludge
pB8	57198	Beta-1	*aadA4*	Biparental	activated sludge bacteria of a waste-water treatment plant
pBB55	73798	Beta-1	*mer* , *qacE* delta, *sul1* , *orf5* , *neo* , *tetAR* , *mph* (E), *msr* (E)	Biparental	manure-treated soil
pDS1	40596	Eta	**no accessory gene**	Triparental(pBBR1MCS)	creek water
pDS3	40806	Beta-1	**no accessory gene**	Triparental(pBBR1MCS)	creek water
pDTC28	61503	Beta-1	*mer* , *bla* $_{GES-5}$, *qacE* delta1, *sul1* , *strAB* , *tetA*	Biparental	River Sediment
pEG1-1	73230	Beta-1	*qacE* , *sul1* , *orf5* , *ANT* (3"), *tetAR*	Biparental	stream sediment
pEMT3	63472	Epsilon	chlorocartechol 1,2-dioxygenase	Biparental	agricultural soil
pG527	80762	Alpha	beta-lacamase, streptomycin, aminoglycoside, bleomycin	Biparental	pig manure
pHH128	56366	Epsilon	*aadA1b* , *catB2*	Biparental	arable soil after application of pig manure
pHH3408	51230	Epsilon	*qacE* delta, *sul1* , *orf5*	Biparental	arable soil 57 days after application of pig manure
pHH3414	55424	Epsilon	*aadA1* , *sul1* , *tetAR*	Biparental	arable soil 85 days after application of pig manure
pKJK5	54383	Epsilon	*aadA11b*	Triparental (pJRD25, IncQ/P-4)	exogenous isolation from soil
pKS208	50604	Gamma	*aphA1-IAB*	Biparental	waste water treatment plant
pKS77	53419	Epsilon	*aadB* , *qacE* delta1, *sul1* , *tetA*	Biparental	arable soil after application of pig manure
pKSP212	54342	Beta-1	*mer* , *sul1* , *aac*	Biparental	hospital waste water treatment plant
pMBUI1	44304	Gamma	**no accessory gene**	Triparental(pBBR1MCS)	University of Idaho arboretum and botanical garden pond
pMBUI8	53313	Beta-1	dihydrolipoamide dehydrogenase	Triparental(pBBR1MCS-5)	creek water

pMCBF6	66615	Zai	*mer* , *mexEFoprN*	Biparental	marine biofilm
pMLUA1	58845	Epsilon	*tetA* , *qacE* delta, *sul1* , *orf5* , *aadA5* , *qacF5* , *mph* (E), *msr* €	Biparental	exogenous isolation from estuarine waters
pMLUA3	57859	Epsilon	*tetA* , *qacE* delta, *sul1,* *orf5* , *aadA4* , *qacF5* , *mph* (E), *msr* (E)	Biparental	exogenous isolation from estuarine waters
pMLUA4	55475	Epsilon	*tetA* , *qacE* delta, *sul1* , *orf5* , *qacF5* , *mph* (E), *msr* (E)	Biparental	exogenous isolation from estuarine waters
pQKH54	69966	Gamma	*mer*	Triparental (pD10)	river epilithon
pRSB223	62099	Beta-2	*oxaA* , *aadA* , *qacE*	Biparental	municipal waste water treatment plant
pRWC72a	61919	Beta-1	*tetA* , *qacE* delta, *sul1* , *mph* (B), *mrx* (B),	Direct capturing	wastewater treatment plant influent
pSN1104-59	50476	Beta-1	**no accessory gene**	Triparental(pBBR1MCS-2)	anaerobic granule
pSP21	72683	Alpha	*aadA1* , *aacA4* , *oxa-2*	Biparental	activated sludge
pTB11	68869	Alpha	*aadA1* , *aadA4* , *tetA*	Biparental	activated sludge basin of a waste-watertreatment plant
pTK9	76102	Beta-1	*mer*	Triparental (IncQ/P-4)	rhizosphere of potato
pTL16	59989	Beta-1	*mer* , *qacE*	Triparental (IncQ/P-4)	rhizosphere of lettuce
pTL21	53599	Beta-1	*mer*	Triparental (IncQ/P-4)	rhizosphere of lettuce
pTL25	102276	Beta-1	*mer* , *siaT*	Triparental (IncQ/P-4)	rhizosphere of lettuce
pTL43	72288	Beta-1	putative D-malate degradative genes	Triparental (IncQ/P-4)	rhizosphere of lettuce
pTL50	39671	Epsilon	**no accessory gene**	Triparental (IncQ/P-4)	rhizosphere of lettuce
pTL52	53601	Beta-1	*mer*	Triparental (IncQ/P-4)	rhizosphere of lettuce
pTL8	51488	Beta-2	**no accessory gene**	Triparental (IncQ/P-4)	rhizosphere of lettuce
pTL9	107234	Beta-1	*mer* , chromosomal DNA	Triparental (IncQ/P-4)	rhizosphere of lettuce
pTP6	54344	Beta-1	*mer*	Triparental (IncQ/P-4)	Mercury-polluted river sediment
pTT11	53011	Beta-1	*mer*	Triparental (IncQ/P-4)	rhizosphere of tomato
pTT25	83086	Beta-1	*qacE*	Triparental (IncQ/P-4)	rhizosphere of tomato
pTT47	43582	Beta-1	**no accessory gene**	Triparental (IncQ/P-4)	rhizosphere of tomato
pTT5	43580	Beta-1	**no accessory gene**	Triparental (IncQ/P-4)	rhizosphere of tomato
pTT60	50519	Delta	*rhaS* (L-rhamnose operon regulatory protein)	Triparental (IncQ/P-4)	rhizosphere of tomato
pWEC911	74056	Alpha	*mer* , tetracycline resistance gene	Biparental	sugar beet rhizosphere

Table S2. IncP-1 plasmids captured from rhizosphere.

Source	Name	No. of plasmid restrition patterns	GenBank accession no.	Size (bp)	IncP-1 supgroup	Insert size including MGEs (bp)	Presense of Tn, integron, and IS elements					Notes for insertions of accessory genes
							Tn*501* (with *mer*)-like	Tn*402*-like	integron (*intlI*)	IS*1071*	others	
potato rhizosphere	pTK9	1	MH392233	76,102	β1	34,364	***trfA-oriV*** **(intact)**	-	-	***trfA-oriV*** **(intact)**	other *tnpA* (*trfA-oriV*)	putative gene sets for catechol and chloroaniline degradation (14,372 bp, *trfA-oriV*)
lettuce rhizosphere	pTL8	1	MH392240	51,448	β2	9,971	-	-	-	***trfA-oriV*** **(intact, with direct repeats)**	Tn*3*-family transposase (*trfA-oriV*)	chlorite dismutase gene in Tn*3*-family transposon (6,625 bp, *trfA-oriV*)<br>hypothetical protein gene (*parA-upf31.0*)
	pTL9	1	MH392241	107,234	β1	58,648 (*trfA-oriV*)<br>6,441 (*traC-parA*)	***trfA-oriV*** **(intact)**	*traC-parA* (remnant)	-	***traC-parA*** **(remnant)**	-	chromosomal DNA region of *Cupriavidus besiliensis* with putative metabolic genes related to glycolate and/or pyruvate (50,420 bp, *trfA-oriV*)<br>molybdate or molybdenum transporter (*modEAGC*) (4,321 bp, *traC-parA*)
	pTL16	1	MH392234	59,989	β1	11,167 (*trfA-oriV*)<br>7,085 (*traC-parA*)	***trfA-oriV*** **[IS*1071* was inserted into *tnpA*$_{Tn501}$ (yielding *tnpA*$_{Tn501}$ Δ)]**	***traC-parA*** **(intact, with class 1 integron)**	***traC-parA*** **(with intact *qacE* gene)**	***trfA-oriV*** **(intact)**	-	-
	pTL21	1	MH392235	53,599	β1	11,439	***trfA-oriV*** **[IS*1071* was inserted into *tnpA*$_{Tn501}$ (yielding *tnpA*$_{Tn501}$ *)]**	-	-	***trfA-oriV*** **(intact)**	-	-
	pTL25	1	MH392236	102,276	β1	59,037	***trfA-oriV*** **[IS*1071* was inserted into *tnpA*$_{Tn501}$ (yielding *tnpA*$_{Tn501}$ Δ)]**	-	-	***trfA-oriV*** **(two IS*1071* remnants)**	IS-like element, several transposase (*trfA-oriV*)<br>IS-like element (IS*116*/IS*110*/IS*902* type transposase) (*klcB-korC*)	Gene sets for putative novel dioxygenase (Rieske-type terminal oxygenase, ferredoxin, ferredoxin reductase for aromatic compound), C4-organic acid transporter, CoA-dependent metabolism (*trfA-oriV*)

	pTL43	1	MH392237	72,288	β1	3,204 (*trfA-oriV*)<br>26,797 (*traC-parA*)	-	***traC-parA*<br>(intact, with metabolic genes and three ISs)**	-	***trfA-oriV*<br>(intact, with direct repeats)**	Three copies of IS*21* family ISs (*traC-parA*)	metabolic genes (extradiol dioxygenase)<br>(*traC-parA*)
	pTL50	4	MH392238	39,671	ε	-	-	-	-	-	-	-
	pTL52	1	MH392239	53,601	β1	11,440	***trfA-oriV*<br>[IS*1071* was inserted into *tnpA* $_{Tn\ 501}$<br>(yielding *tnpA* $_{Tn501}$*)]**	-	-	***trfA-oriV*<br>(intact, with direct repeats)**	-	-
Tomato rhizosphere	pTT5	3	MH392245	43,580	β1	2,523	-	-	-	*traC-upf31.0*<br>(remnant, *parA* was lost)	-	-
	pTT11	21	MH392242	53,011	β1	11,168	***trfA-oriV*<br>[IS*1071* was inserted into *tnpA* $_{Tn\ 501}$<br>(yielding *tnpA* $_{Tn501}$Δ)]**	-	-	*trfA-oriV*<br>(*tnpA* $_{IS1071}$ was frameshifted)	-	-
	pTT25	1	MH392243	83,086	β1	38,998	-	*traC-parA*<br>(remenant, *tniR* $_{Tn402}$ was disrupted by IS*3* family element, *tniAB* $_{Tn402}$ and partial *tniQ* $_{Tn402}$ were deleted)	***traC-parA*<br>(with intact *qacE* gene)**	***traC-parA*<br>(three copies of intact IS*1071*, two of them were identical, the other showed one nucletide substitution)**	-	acetyl-coenzyme A synthetase, putative genes for transporter, transcriptional regulator, duplicated DNA regions surrounded by IS*1071*
	pTT47	12	MH392244	43,582	β1	2,524	-	-	-	*traC-upf31.0*<br>(remnent, *parA* was lost)	-	-
	pTT60	2	MH392246	50,519	δ	9,178	-	***traC-parA*<br>(intact, with DRs)**	-	-	-	Tn*402* -like transposon with genes for putative ester cyclase, and glucose-6-phosphate isomerase

Soil (ammended with manure)	pBB55	1	MH392232	73,798	β1	25,470 (*trfA-oriV*)<br>7,745 (*traC-parA*)	***trfA-oriV*** **[IS*1071* Tn*3*-family transposon and composite transposon were inserted into *tnpA* $_{Tn501}$ (yielding *tnpA* $_{Tn501}$Δ)]**	*parA-upf31.0* (remnant, only one copy of the inverted repeats of Tn*402* was found, *tniABRQ* $_{Tn402}$ were deleted by insertion of IS*1326* family elementno other genes)	***parA-upf31.0*** **(with *qacHa*, *qacHb*, *bla*, *qacEsul1orf5*)**	***trfA-oriV*** **(intact)**	IS*1326-like* (*parA-upf31.0*)<br><br>Tn*3* family transposon (remnant) (*trfA-oriV*)	antibiotic resistance genes, *tet*, gene for APH(3") family aminoglycoside O-phosphotransferase (not named yet), *strB*, in Tn*501*-like transposon remnant, and *qacL_1*, *qacL_2*, and *bla* in Tn*402*-class1 integron

Table S3. Accession no. of IncP-1 plasmids.

Name	Accession number	Organism
unnamed5	NZ_CP038640.1	*Cupriavidus oxalaticus*
unnamed2	NZ_CP019238.1	*Rhodoferax koreense*
unnamed_2	NZ_CP015373.1	*Pandoraea pnomenusa*
RK2	BN000925.1	Birmingham IncP-alpha plasmid
R906	KF743818.1	*Bordetella bronchiseptica*
R772	KF743817.1	*Proteus mirabilis*
R751	NC_001735.4	*Klebsiella aerogenes*
pZJ623	KX580713.1	*Escherichia coli*
pYS1	NC_019369.1	*Burkholderia cepacia*
pYDC107_41	NZ_CP025711.1	*Escherichia coli*
pWEC911	JX469833.1	uncultured bacterium
pWDL7	GQ495894.1	synthetic construct
pUO1	NC_005088.1	*Delftia acidovorans*
pTT60	MH392246.1	uncultured bacterium
pTT5	MH392245.1	uncultured bacterium
pTT47	MH392244.1	uncultured bacterium
pTT25	MH392243.1	uncultured bacterium
pTT11	MH392242.1	uncultured bacterium
pTP6	AM048832.1	uncultured bacterium
pTL9	MH392241.1	uncultured bacterium
pTL8	MH392240.1	uncultured bacterium
pTL52	MH392239.1	uncultured bacterium
pTL50	MH392238.1	uncultured bacterium
pTL43	MH392237.1	uncultured bacterium
pTL25	MH392236.1	uncultured bacterium
pTL21	MH392235.1	uncultured bacterium
pTL16	MH392234.1	uncultured bacterium
pTK9	MH392233.1	uncultured bacterium
pTB30	NC_016968.1	*Comamonas testosteroni*
pTB11	AJ744860.1	uncultured bacterium
pSP21	CP002153.1	uncultured bacterium
pSN1104-59	AP018709.1	uncultured bacterium
pSEHO0A1	AGRM01000006.1	*Salmonella enterica* subsp. *houtenae*
pRWC72a	JX486125.1	uncultured bacterium
pRSB223	JX469828.1	uncultured bacterium
pQKH54	AM157767.1	uncultured bacterium
pP09019	NZ_CP017455.1	*Dickeya solani*
pNCYU-29-69-3_MCR3	CP042602.1	*Escherichia coli*
pNB8c	NC_019264.1	*Delftia acidovorans*
pMLUA4	KC964607.1	uncultured bacterium
pMLUA3	KC964606.1	uncultured bacterium
pMLUA1	KC964605.1	uncultured bacterium
pMCR3_WCHEC-LL123	MF489760.1	*Escherichia coli*
pMCR3_WCHEC1943	MF678351.1	*Escherichia coli*
pMCR3_025943	NZ_CP027203.2	*Escherichia coli*
pMCR16_P053	KY352406.1	*Salmonella enterica* subsp. *enterica* serovar Typhimurium
pMCR_WCHEC1622	KY463452.1	*Escherichia coli*
pMCR_BE2-5	NZ_CP032987.1	*Escherichia coli*
pMCR_1511	KX377410.1	*Klebsiella pneumoniae*
pMCBF6	NC_025028.1	uncultured bacterium
pMBUI8	KC170279.1	uncultured bacterium
pMBUI1	JQ432563.1	uncultured bacterium
pLME1	NC_019263.1	*Delftia acidovorans*
pLM159.2	NZ_CP018471.1	*Xanthomonas vesicatoria*
pKV29	NC_019312.1	*Delftia* sp. KV29
pKSP212	JX469831.1	uncultured bacterium

pKS77	JQ004409.1	uncultured bacterium
pKS208	JQ432564.1	uncultured bacterium
pKPCAPSS	KP008371.1	*Klebsiella pneumoniae*
pKJK5	AM261282.1	uncultured bacterium
pKJK172	NZ_CP028340.1	*Thauera aromatica* K172
pJP4	NC_007337.1	*Cupriavidus pinatubonensis* JMP134
pIJB1	NC_019378.1	*Burkholderia cepacia*
pICP-4GES	MH053445.1	*Pseudomonas aeruginosa*
pI2	NC_016978.1	*Comamonas testosteroni*
pHS102707	NC_023907.1	*Escherichia coli*
pHNGDF36-1	MF978389.1	*Escherichia coli*
pHNGDF1-1	MF990207.1	*Escherichia coli*
pHNFP671	KP324830.1	*Escherichia coli*
pHKSHmcr1_P2_p1	MF136778.1	*Escherichia coli*
pHH3414	JQ004408.1	uncultured bacterium
pHH3408	JQ004407.1	uncultured bacterium
pHH128	JQ004406.1	uncultured bacterium
pHB44	KU356988.1	*Variovorax paradoxus*
pG527	JX469830.1	uncultured bacterium
pEST4011	NC_005793.2	*Achromobacter denitrificans*
pENH91	NZ_CP017760.1	*Cupriavidus necator*
pEN3600	NZ_CP035638.1	*Enterobacter cloacae*
pEMT3	JX469827.1	uncultured bacterium
pEGD-AKN5	KP289281.1	*Pseudomonas* sp. EGD-AKN5
pEG1-1	MG879028.1	uncultured bacterium
pECSC102	MG552133.1	*Escherichia coli*
pDTC28	KU238092.1	uncultured bacterium
pDS3	JX469834.1	uncultured bacterium
pDS1	KC170283.1	uncultured bacterium
pDB1	NC_019320.1	*Variovorax* sp. DB1
pCNB	NC_010935.1	*Comamonas testosteroni* CNB-1
pCFSA664-2	NZ_CP033354.2	*Salmonella enterica* subsp. *enterica*
pC1-1	NC_019283.1	*Delftia acidovorans*
pBS64	KU356987.1	*Variovorax paradoxus*
pBS228	NC_008357.1	*Pseudomonas aeruginosa*
pBRA100	NC_017908.2	*Mycobacteroides abscessus* subsp. *bolletii* F1725
pBP136	NC_008459.1	*Bordetella pertussis*
pBII_1	NZ_CP009797.1	*Burkholderia ambifaria* AMMD
pBHB	NC_021077.1	*Comamonas* sp. 7D-2
pBB55	MH392232.1	uncultured bacterium
pBAM1	NZ_CP028967.1	*Aminobacter* sp. MSH1
pB8	AJ863570.1	uncultured bacterium
pB5	CP002151.1	uncultured bacterium
pB4	AJ431260.1	uncultured bacterium
pB3	AJ639924.1	uncultured bacterium
pB12	JX469826.1	uncultured bacterium
pB11	CP002152.1	uncultured bacterium
pB1	JX469829.1	uncultured bacterium
pAOVO02	NC_008766.1	*Acidovorax* sp. JS42
pAMMD1	NC_008385.1	*Burkholderia ambifaria* AMMD
pALIDE02	NC_014911.1	*Alicycliphilus denitrificans* BC
pAKD4	GQ983559.1	uncultured bacterium
pAKD34	JN106175.1	uncultured bacterium
pAKD33	JN106174.1	uncultured bacterium
pAKD31	JN106173.1	uncultured bacterium
pAKD29	JN106172.1	uncultured bacterium
pAKD26	JN106171.1	uncultured bacterium
pAKD25	JN106170.1	uncultured bacterium
pAKD18	JN106169.1	uncultured bacterium
pAKD17	JN106168.1	uncultured bacterium
pAKD16	JN106167.1	uncultured bacterium

pAKD15	JN106166.1	uncultured bacterium
pAKD14	JN106165.1	uncultured bacterium
pAKD1	JN106164.1	uncultured bacterium
pADP-1	NC_004956.1	*Pseudomonas* sp. ADP
pAAA83	NC_024998.1	*Acidovorax avenae* subsp. *avenae*
pA81	NC_006830.1	*Achromobacter xylosoxidans* A8
pA22732-IMP	KJ588780.1	*Achromobacter xylosoxidans*
pA1	NC_007353.2	*Sphingomonas* sp. A1
p712	NC_019318.1	*Ralstonia pickettii*
p2-T1	NZ_CP021650.1	*Acidovorax* sp. T1
p14EC001a	NZ_CP024128.1	*Escherichia coli*
